# Mapping HIV-1 Vaccine Induced T-Cell Responses: Bias towards Less-Conserved Regions and Potential Impact on Vaccine Efficacy in the Step Study

**DOI:** 10.1371/journal.pone.0020479

**Published:** 2011-06-10

**Authors:** Fusheng Li, Adam C. Finnefrock, Sheri A. Dubey, Bette T. M. Korber, James Szinger, Suzanne Cole, M. Juliana McElrath, John W. Shiver, Danilo R. Casimiro, Lawrence Corey, Steven G. Self

**Affiliations:** 1 Vaccine and Infectious Disease Division, Fred Hutchinson Cancer Research Center, Seattle, Washington, United States of America; 2 Vaccine Basic Research Department, Merck Research Laboratories, West Point, Pennsylvania, United States of America; 3 Department of Medicine, University of Washington, Seattle, Washington, United States of America; 4 Department of Biostatistics, University of Washington, Seattle, Washington, United States of America; 5 Department of Laboratory Medicine, University of Washington, Seattle, Washington, United States of America; 6 Department of Epidemiology, University of Washington, Seattle, Washington, United States of America; 7 Santa Fe Institute, Santa Fe, New Mexico, United States of America; 8 Los Alamos Laboratory, Los Alamos, New Mexico, United States of America; University of Palermo, Italy

## Abstract

T cell directed HIV vaccines are based upon the induction of CD8+ T cell memory responses that would be effective in inhibiting infection and subsequent replication of an infecting HIV-1 strain, a process that requires a match or near-match between the epitope induced by vaccination and the infecting viral strain. We compared the frequency and specificity of the CTL epitope responses elicited by the replication-defective Ad5 gag/pol/nef vaccine used in the Step trial with the likelihood of encountering those epitopes among recently sequenced Clade B isolates of HIV-1. Among vaccinees with detectable 15-mer peptide pool ELISpot responses, there was a median of four (one Gag, one Nef and two Pol) CD8 epitopes per vaccinee detected by 9-mer peptide ELISpot assay. Importantly, frequency analysis of the mapped epitopes indicated that there was a significant skewing of the T cell response; variable epitopes were detected more frequently than would be expected from an unbiased sampling of the vaccine sequences. Correspondingly, the most highly conserved epitopes in Gag, Pol, and Nef (defined by presence in >80% of sequences currently in the Los Alamos database www.hiv.lanl.gov) were detected at a lower frequency than unbiased sampling, similar to the frequency reported for responses to natural infection, suggesting potential epitope masking of these responses. This may be a generic mechanism used by the virus in both contexts to escape effective T cell immune surveillance. The disappointing results of the Step trial raise the bar for future HIV vaccine candidates. This report highlights the bias towards less-conserved epitopes present in the same vaccine used in the Step trial. Development of vaccine strategies that can elicit a greater breadth of responses, and towards conserved regions of the genome in particular, are critical requirements for effective T-cell based vaccines against HIV-1.

**Trial Registration:**

ClinicalTrials.gov NCT00849680, A Study of Safety, Tolerability, and Immunogenicity of the MRKAd5 Gag/Pol/Nef Vaccine in Healthy Adults.

## Introduction

In principle, an effective HIV-1 vaccine designed to elicit antiviral T cell immunity must direct those responses to T cell epitopes likely to be present in the diverse population of circulating viral strains. HIV-1 vaccines must either generate sufficiently broad responses (multiple or cross-reactive responses) to compensate for the extensive sequence variability among circulating HIV-1 strains, or include highly-conserved epitopes likely to be present in a high percentage of strains.

The HIV-1 genes represented in most candidate HIV vaccines are typically full-length (or nearly full-length) genes derived from natural isolates. These can be based on a natural strain specifically selected to be relatively central to circulating strains in a given population, to maximize the potential cross-reactivity [1], or by selecting a natural sequence that maximizes coverage of potential epitopes in a population of sequences [2], [3]. Alternatively, one can use synthetic sequences (e.g., consensus or inferred ancestral [4], [5], [6]), or sets of sequences designed to optimize potential epitope coverage as the basis for antigen design [2], [7]. No studies have determined experimentally whether conserved or variable epitopes are preferentially recognized by the human T-cells after vaccination with such immunogens and how this preference is related to the outcome of clinical vaccine trials. Here, we present the results of an extensive epitope mapping study from healthy volunteers who received the same vaccine candidate as was subsequently administered to high-risk volunteers in a Phase IIB vaccine trial. The epitope mapping is supplemented with theoretical analyses to evaluate the frequency and breadth of T cell response and evaluate the distribution of highly vs. poorly conserved epitopes. This leads to conclusions for potential impact on vaccine efficacy and strategies for future HIV vaccine development.

The Step trial, a test of concept study of a T cell based vaccine, failed to reach its interim criteria for reduction in infection rates and/or reduction of set point viremia, and further vaccinations were suspended in September 2007 [8], [9]. The vaccine candidate, developed by Merck Research Laboratories, consisted of a mixture of replication-defective Adenovirus type 5 constructs containing the HIV-1 *gag, pol,* and *nef* genes which were inserted into the E1 region of the Ad5 backbone. *Gag* and *pol* were selected because of their relatively high conservation both interclade and intraclade among all HIV gene products. Specifically, the *gag* coding sequence was derived from the CAM-1 strain of HIV-1 (GenBank Locus BAA00992) [10], because its Gag amino acid sequence most closely resembled the clade B consensus. The *pol* construct included only the reverse transcriptase and integrase gene products from IIIb, and *nef* from JRFL strains. All three transgenes were optimized by using frequently utilized codons to improve protein expression in mammalian cells [11]. The *pol* transgene segment was inactivated by substituting alanine codons for amino acids at enzymatically active sites [11], [12], [13], [14], [15], [16], [17], [18], [19], and the *nef* transgene segment was inactivated through substitutions that prevent attachment to the cytoplasmic membrane and retrotrafficking into endosomes [17], [20]. The vaccine was formulated as a 1∶1∶1 mixture of each gene product and 3 separate doses of 10^10^ viral particles of the mixture were administered. The current study parallels the Step trial; the vaccine was immunogenic in most individuals: >70% of subjects who received the vaccine in the Step trial had detectable HIV-specific CD8+ T lymphocytes that were capable of producing multiple cytokines, including IFN-γ and TNF-α; these responses were long-lasting, persisting for more than one year [9]. Despite these immune responses, the trial failed to meet its endpoints for reduction in acquisition or reducing post-acquisition viral load. The reasons behind the lack of efficacy despite being highly immunogenic remain unclear and are central to the future development of an effective HIV vaccine.

We undertook an analysis of whether the T-cell epitope response specificities elicited by vaccination were within conserved or variable regions of the HIV proteins and whether the vaccine elicited the type of T cell coverage likely to be effective after encountering the diversity of HIV-1 circulating in the regions where the vaccine trial was conducted. Specifically, we compared the overall conservation levels of observed epitopes to 1) all the 9-mer CTL epitopes within the three immunogens reported in the LANL database (“known epitopes”) and 2) all the possible 9-mers (we defined as potential T cell epitopes [PTEs]) within the three immunogens.

## Results

### T-cell epitope response breadth

We performed epitope mapping on 72 subjects who participated in an earlier Phase I trial of the same vaccine utilized in the Step study. Among them, 43 subjects were Ad5 seronegative and 29 were Ad5 seropositive at enrollment. The median age was 35; 85% were Caucasian; 68% were male and 32% female. Their responses to vaccination were similar if not higher in magnitude to those enrolled in the Step trial. Details of the T cell and antibody responses to the vaccine among these subjects have been described [17]. Moreover, the subjects we analyzed were enrolled from many US study sites in which the ensuing Step trial was conducted. The incidence of HIV-1 acquisition in Step was highest in the U.S [8]. As such, the epitope mapping data of these samples from the earlier Phase I trial are of direct relevance to immune responses seen in Step.

All vaccinees evaluated had positive responses to the Gag 15-mer peptide pool (72/72) and most also had responses to Nef 15-mer (68/72) and/or Pol 15-mer (56/72). Subsequent mapping of the positive protein response using 9-mer minipools demonstrated that CD8+ T cell epitopes were recognized across the length of all three proteins with detectable responses to one or more minipool per protein in 54% of Gag, 56% of Nef, and 79% of Pol mapped individuals (**Table 1**). A relatively high number of minipools per subject were detected in Pol; 20% of mapped individuals had 4 or more detectable Pol minipool responses, while only 3% of Gag and 3% of Nef had equally broad responses. The median (and mean) of positive minipool responses per subject was 1 (1) for Gag, 1 (1) for Nef and 2 (3) for Pol. Although 15–22% of minipools were recognized by only a single subject, more minipools (23% in Gag, 50% in Nef and 42% in Pol) were recognized by two or more subjects and response rates to some individual minipools were relatively high, with 6–10 subjects responding to a common minipool (**Table S1**). Deconvolution of the individual 9-mer peptide response within the positive minipools was completed for 228 minipools where response magnitude and sample supply were sufficient. Individual 9-mer responses were undetectable or below the positivity criteria in 61 of the deconvoluted minipools. Responses to a single 9-mer epitope per minipool were detected from 84% of the minipools. Responses to two, or rarely three, 9-mer epitopes were detected from the remaining 16% of minipools, but most were offset by just 1 or 2 amino acids with one dominant response; thus, these were likely responses to the same epitope. A total of 105 distinct epitopes were detected from the positive minipools, with 30, 20, and 55 in Gag, Nef, and Pol, respectively (**Table 2**). The 9-mer peptides that induced the most frequent responses (≥5 subjects) were Gag-RLRPGGKKK, Gag-ATLYCVHQK, Nef-RVRRTEPAA, Nef-AVDLSHFLK and Pol-ITTESIVIW. Approximately half (57 of 105) of the gene-specific CTL responses detected in vaccinees were directed at epitopes unique to an individual vaccinee (20 in Gag, 9 in Nef, 28 in Pol).

**Table 1:** Breadth of response: Number of positive minipool responses among Merck Ad5 *gag/pol/nef* vaccine responders.

No. positive pools (epitopes)	Gag^a^	Pol	Nef
0 pools	33 (46%)	12 (21%)	30 (44%)
1 pool	25 (35%)	15 (27%)	26 (38%)
2 pools	8 (11%)	9 (16%)	8 (12%)
3 pools	3 (4%)	9 (16%)	2 (3%)
≥4 pools	3 (4%)	11 (20%)	2 (3%)
N mapped	72	56	68

a Number (%) of vaccinees.

**Table 2:** Frequency of distinct epitope responses among Merck Ad5 *gag/pol/nef* vaccine responders.

No. vaccinees responding to the same epitope	Gag	Pol	Nef
1	20	28	9
2–3	6	24	7
4–5	2	3	4
≥6	2	0	0
Total distinct epitopes observed	30	55	20

### T-cell epitope distribution along the immunogens

**Figure 1:**
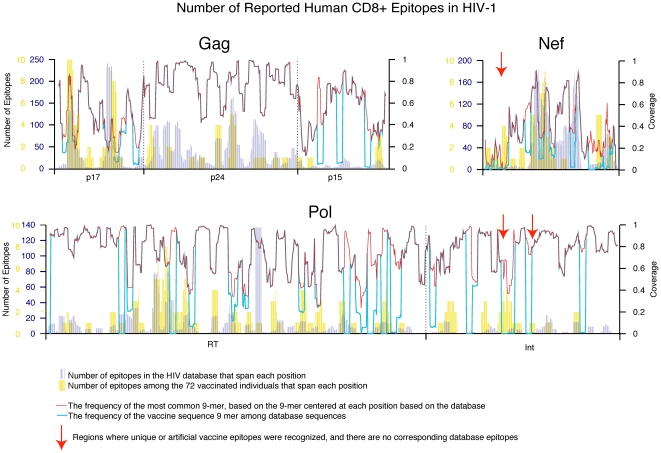
Tracking the frequency of defined epitopes to the vaccine proteins and the database of responses to natural infection and across the vaccine proteins. The blue histograms mark the number of epitopes in the database that span each amino acid position in the protein. The yellow bars track the same number for the 72 vaccinees. The bright blue line tracks the level of coverage the vaccine provides of B clade sequences in the database. The sharp dips are either a consequence of the natural strain carrying very unusual amino acids, or amino acids engineered into the protein enhance the safety of the vaccine. The red line, in contrast, is a “neutral” measure of the overall diversity of each 9-mer, not relative to any one strain; it tracks the frequency of the most common 9-mer, for each 9-mer. Frequency of 9-mer PTEs is calculated from Los Alamos database sequences to reflect contemporary natural infections as supported by the high correlation between them (Figure S1).

Vaccinated subjects with similar HLA types exhibited markedly different epitope-specific responses. Despite individual variability in the epitope specificity of the vaccine induced T cell response, the location of the epitope responses after vaccination along the protein sequence tracked well with the distribution of responses reported in the literature after natural infection (**Figure 1**). There was a very strong correlation between the number of epitopes that span each position in the protein among the vaccinees and the reported human HIV epitopes in the Los Alamos database (Kendall's rank correlation p<2×10^−16^). This consistency suggests that the localization of responses to the vaccine follows that of initial infection, a desirable vaccine characteristic as the vaccine elicited responses targeted to epitopes that are processed and presented in infected human subjects. Given the relative length of the Nef protein (216 amino acids, versus 850 in Pol and 500 in Gag), there was a significantly greater density of responses to Nef than to the other proteins (Chi Square test, p = 0.03 comparing Nef and Gag, p = 0.007 comparing Nef and Pol).

### Vaccine epitope coverage

We next performed a series of analyses to evaluate the likelihood vaccinations would elicit responses to epitopes likely to be present in circulating strains of HIV-1, a concept termed “coverage.” A valid question for coverage analysis is whether the sequences from Los Alamos database are representative of contemporary natural infections. Frequency comparison of PTEs derived from contemporary, incident HIV infections with those from Los Alamos database shows excellent correlation (**Figure S1**, p<0.0001), which supports the use of database sequences in this analysis. We define vaccine coverage here to mean the fractions of viral sequences in the target population containing ≥1, ≥2, ≥3,… of the epitopes for a given vaccinee; the average of each of these quantities over a sample of vaccinees provides the expected vaccine coverage at a given degree (e.g., expected coverage by ≥3 epitopes). We note that for a genetically homogeneous viral population, the expected coverage corresponds to response breadth (e.g., fraction of vaccinees with ≥3 epitopes). Thus, the expected coverage is a combination of both the breadth of response and the pattern of population frequencies for the sets of epitopes among vaccinees. Direct estimates of expected vaccine coverage are presented in **Table 3**. The average vaccinee would cover with ≥1 epitopes in any gene 57% of a clade B viral population and this coverage decreases to 35% for ≥2 epitopes and 20% with ≥3 epitopes.

**Table 3:** Expected vaccine coverage: Fraction of target viral sequences covered by epitopes among recipients of Merck Ad5 *gag/pol/nef* vaccine.

No. Epitopes	Any Gene^a^	Gag	Pol	Nef
0 epitopes	17%	16%	10%	35%
1 epitope	22%	25%	21%	10%
2 epitopes	15%	5%	11%	1%
3 epitopes	10%	1%	5%	0%
≥4 epitopes	10%	0%	11%	0%

a Expected % of viral population covered.

Our analysis also revealed a low frequency of recognized epitopes in the circulating strains that is not obvious from protein similarity (or distance) (**Table S2**). For example, the Nef insert has an average protein similarity of 78% to subtype B strains while the average epitope frequency in the same viruses is only 9%. This is a direct consequence of CD8 epitopes typically being around 9 amino acids long; if 22% of the positions vary between two strains it is correspondingly highly likely that one or more of the 9 positions in the CD8 epitope will vary.

### Variable epitopes are preferentially recognized

**Figure 2:**
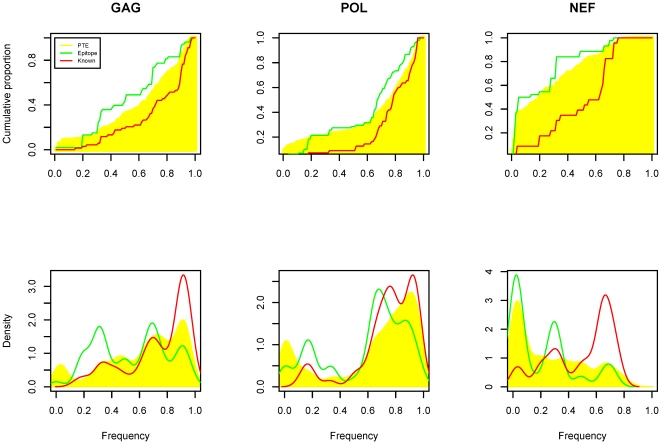
Comparison of cumulative (upper) and density (lower) distributions of PTE, known and defined epitope in subtype B strains. The yellow shaded area indicates the cumulative and density distributions of PTEs from vaccine inserts. The green and red lines are the distribution curves for defined epitope and known epitopes respectively. The relative variable epitopes are enriched and highly conserved epitopes are underrepresented in this study. Known epitopes in LANL are overrepresented as expected.

To augment this analysis of expected vaccine coverage, we explicitly examined epitope-specific coverage by comparing the overall conservation levels of observed epitopes to 1) known epitopes within the three immunognes and 2) all the 9-mers within the three immunogens in subtype B strains (**Figure 2**). The frequencies of the mapped epitopes among vaccinees to each of the three proteins, Gag, Pol, and Nef are significantly lower than those of the known epitopes (Kolmogorov-Smirnov test; p<0.001 Gag, p<0.001 Nef, p<0.01 Pol), although this result is likely due to an artifact of the over-representation of conserved elements in peptides used to detect CTL responses (the “founder effect”) [21], [22]. This effect is particularly pronounced for the Nef protein. Recognized epitope frequencies to the three HIV proteins after vaccination were also lower than 9-mer PTEs in the immunogens (Kolmogorov-Smirnov test; p<0.05 Gag, p<0.05 Nef, p<0.01 Pol), suggesting a preference for recognition of variable epitopes. Although statistically significant for Gag and Pol, the magnitude of these differences is relatively modest (**Figure 2**). Nonetheless, detailed examination of the differences for Gag and Pol suggest that relatively variable epitopes (with frequency 0.1–0.4) were over-represented in the observed epitopes (**Figure 2**, **Table 4**) and highly conserved epitopes (frequency >0.8) in Gag and Pol were under-represented. A similar difference in the distribution of recognition frequencies of highly conserved versus less conserved potential epitopes after wild-type infection with HIV-1 was noted based on the Los Alamos Immunology database (Wilcoxon rank test p = 0.0006), suggesting some form of “antigenic masking” of these regions may occur.

**Table 4:** Comparison of PTE and defined epitope with different conservation level.

Conservation level	Gag		Pol		Nef	
	Epitope	PTE	Epitope	PTE	Epitope	PTE
Highly variable(0-)	1 (2%)	43 (9%)	6 (6%)	132 (16%)	22 (50%)	81 (39%)
Relatively variable(0.1-)	18 (34%)	62 (13%)	21 (21%)	64 (8%)	15 (34%)	63 (30%)
Relatively conserved(0.4-)	24 (45%)	228 (46%)	45 (46%)	286 (34%)	7 (16%)	61 (29%)
Highly conserved(0.8-)	10 (19%)	159 (32%)	26 (27%)	360 (43%)	0 (0%)	3 (1%)
**Total**	**53** (100%)	492 (100%)	**98** (100%)	842 (100%)	**44** (100%)	208 (100%)

**Figure 3:**
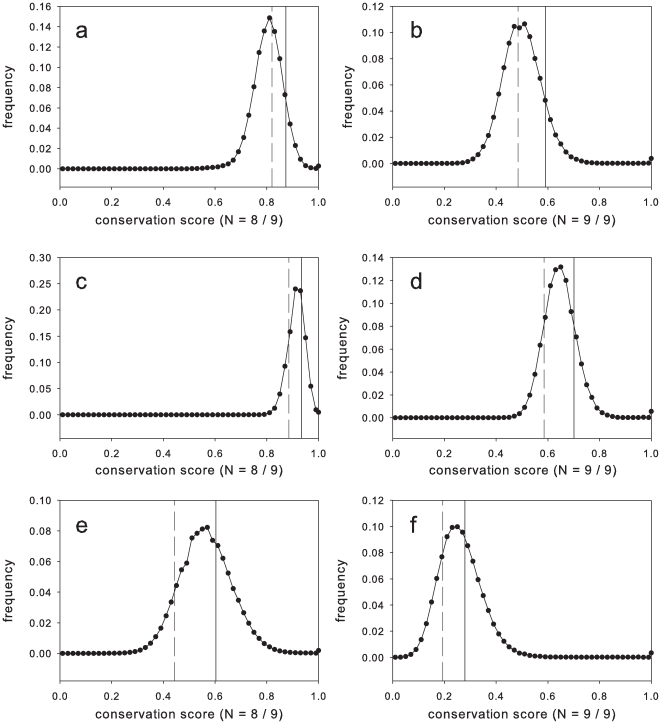
Histograms of all possible N-mer conservation scores for Gag (a) N = 8/9, (b) N = 9/9; Pol (c) N = 8/9, (d) N = 9/9; and Nef (e) N = 8/9, (f) N = 9/9. Solid vertical lines show the mean conservation of the Merck vaccine sequence vs. all others in the distribution. Dashed vertical lines show the mean conservation score of the ELISpot response.

Compared to other subtype B viral strains, the Merck sequences perform better as vaccine sequences than most subtype B, due to being selected to be relatively central relative to circulating strains (**Figure 3**). This mitigates against the bias towards less-conserved epitopes. Comparing against the predicted overlap with the epitopes directly identified in the 9-mer epitope mapping data, these opposing effects nearly cancel so that the mean conservation score of a mapped epitope is nearly equal to the typical clade B epitope for Gag. That is, the bias for less-conserved epitopes is cancelled by the highly-conserved Gag CAM1 sequence. The same opposition holds for Pol and Nef, except that the net effect is a mean epitope conservation of less than the typical clade B epitope. For Pol and Nef, there is a 3–5% and 11–6% respective additional risk of a mismatch, depending upon the particular assumption that 8 or 9 amino acids in a 9-mer must match (**Table 5**).

**Table 5:** Comparison of 9-mer overlap of vaccine sequences, mapped epitopes with clade B sequences.

Gene	N = 8/9 amino acid identity expectation value			N = 9/9 amino acid identity expectation value		
	Intra-Clade B	vaccine sequence	mapped 9-mers	Intra-Clade B	vaccine sequence	mapped 9-mers
Gag	81%	87%	82%	49%	59%	49%
Nef	55%	60%	44%	25%	28%	19%
Pol	92%	93%	89%	64%	70%	59%

### Optimal T-cell epitope coverage

**Figure 4:**
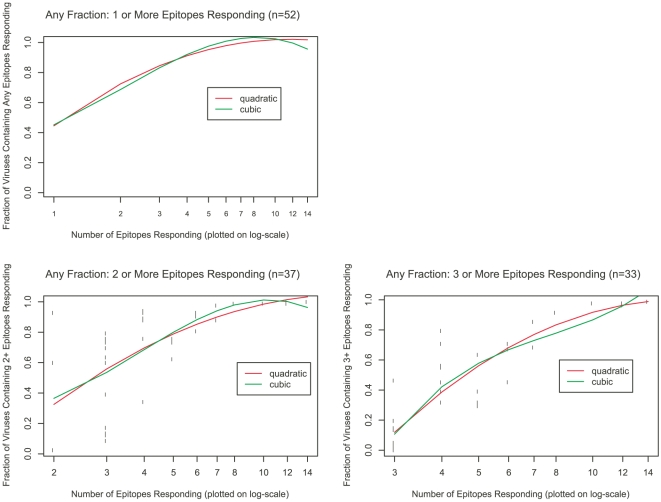
Coverage by ≥1, ≥2 and ≥3 epitopes of a Subtype B viral population vs response breadth (number of epitopes). The points are subject-specific estimates of % viral population (Subtype B) covered by ≥1, ≥2 and ≥3 epitopes versus subject-specific breadth (epitope number) on log scale. The data fit well with both quadratic and cubic regression.

We then performed a simple statistical analysis of the T cell breadth required to achieve a high probability that vaccine elicited epitope responses will be of the type that match the epitope frequency in circulating strains in a population. We plotted the individual-level vaccine coverage probabilities versus breadth of response (number of defined epitopes) and fit non-linear quantile regression models to the resulting scatterplot in order to project the response breadth required for different levels of expected vaccine coverage (**Figure 4**). The results of these analyses suggest that a T cell based vaccine containing gag/pol/nef would require four or more epitope responses to be elicited in order to expect 90% coverage by 1 or more epitopes. As epitope escape early in infection has been reported, it is desirable to have multiple epitopes present; our analysis indicates that two or more epitopes at 90% coverage need 6 or more epitopes to be recognized, and three or more epitopes need 8 to 9 epitopes. This prediction assumes the same degree of conservation of epitopes as observed in the current data. However, if responses to more highly conserved epitopes are elicited by vaccination, the number of epitopes required to achieve coverage may be markedly reduced. Development of a more detailed predictive model of vaccine coverage as a function of both breadth and epitope-level frequency would be useful for guiding future insert designs and for assessment of coverage by future candidate vaccines. The above analysis of epitope coverage implicitly assumes that T-cell epitopes have equal efficacy. For example, recent studies suggest that epitopes restricted by some HLA alleles are more likely to be associated with reduced viral load than other epitopes [23], [24].

## Discussion

Our analyses provide an important series of observations about the outcome of the Step trial and future immunogen design. It is clear that while immunogenic in most individuals, the Merck gag/pol/nef vaccine tended to elicit responses to fewer highly conserved epitopes and more less-conserved epitopes than would be predicted from the vaccine sequences alone. An interesting question is raised as to how generic this bias towards less-conserved epitopes may be. The mechanism for these observations are unclear; potential explanations include that such highly conserved but relatively immunologically silent regions could be the consequence of retroviruses evolving to evade epitope processing in regions where mutations have a very high fitness cost, or could be due to very conserved domains also being conserved in HERVs (Human Endogenous Retroviruses), causing such regions to be seen as “self”. To our knowledge, this is the first study to analyze this question in the context of high-resolution epitope mapping with a high number of patients and a clinical HIV-1 candidate. The extent to which the bias may be related to this vaccine candidate, choice of viral vector delivery, or vaccination vs. natural infection is an important question to guide further vaccine development.

We consider the implications of these results upon vaccine efficacy in general, and the Step trial (which employed the same vaccine as studied here) in particular. First, patients became infected in both placebo and vaccine arms of the Step trial. Mutation of HIV-1 to escape effective T cell recognition has been frequently observed in humans and non-human primates. The probability of escape is a complicated function of viral load, conservation at the amino acid and nucleotide level, and HLA association. Analyses of these complex interactions through HIV-1 sequencing of infected patients are underway. We merely point here out that eliciting responses to highly-conserved epitopes would, in general, be expected to reduce the overall frequency of viral escape. Second, the number of persons who become infected may be greater for a T cell vaccine that elicits responses to less-conserved epitopes. Less conserved epitopes are less likely to be shared between vaccine and infection virus strains. Assuming a worst-case complete immunodominance model such that only a single epitope amongst Gag, Pol, or Nef is recognized (despite our data to the contrary in **Table 3**), and if failure to match this single epitope leads to infection, what would be the potential implications on predicted infection rates?

The experiments and analyses are based upon epitopes of only 9 amino acids in length, thereby omitting CD8 epitopes of 8, 10 or 11 amino acids. In this and subsequent analyses, we may undercount these less frequent non-9-mer epitopes. For example, epitope mapping of select vaccinees with B57/B58 alleles and B27 alleles has shown that key 10 mer Gag epitopes such as TW10 and KK10, respectively, can be detected with peptides 10 or 15 amino acids in length but not with 9-mer peptides. Undercounting of vaccine-induced epitopes may also occur due to the ELISpot assay method. Positivity criteria were established to limit false positive responses to 5% or less, yet some real but weaker responses may be missed at these cut-offs, particularly as responses are narrowed down to the single peptide level. A second issue may complicate the interpretation of the results is TCR recognition degeneracy; A study of 9-mer ELISpot mapping data and multi-isolate sequencing from HIV-infected individuals [25] indicated that one mismatch per 9-mer can still be cross-reactive (although this is subject to HLA restriction). However, even with this cross-reactivity suggested by experiment, the requirement that multiple amino acids match elsewhere in the 9-mer leads to a nonlinear relationship between the number of 9-mers matching and the number of amino acids [25]. Specifically, as homologies move from more to less conserved, epitope overlap will fall off faster than a traditional sequence/protein homology score would suggest, and this disparity is apparent in actual epitope mapping data. This is a further consideration in the types of inserts that should be designed for candidate HIV-1 vaccines [2], [26], [27]. Another finding worth noting from this study is that vaccinated subjects with similar HLA types exhibited markedly different epitope-specific responses, which emphasizes the importance of other factors other than HLA restriction in immune recognition. Indeed, we observed a strikingly different response pattern on the vaccine participants of STEP trial (data not shown).

Our analysis illustrates the need to explore several new avenues for eliciting effective T cell-based immunity for HIV-1 vaccines. One approach to achieving the immune focusing required to elicit responses to conserved regions of the genome is to construct artificial immunogens by linking together highly-conserved epitopes or regions [28]; however, such strategies have proved poorly immunogenic in human phase I immunogenicity trials [29], [30]. Alternatively, one could attempt to develop immunogens with variable regions masked against immune recognition. With either of these approaches, a vaccine that stimulated responses primarily to these most-conserved epitopes may be less effective if these epitopes are infrequently presented in natural infection. Alternatively, algorithms could be designed to choose optimal natural sequences, or sets of natural sequences, to maximize epitope breadth and conservation. These could be extended to heterologous inserts for prime and boost vaccination to successively boost the conserved epitope determinants. This strategy, especially if utilized with one or more viral vectors, may generate responses focused on more highly conserved epitopes with each successive inoculation, while shared conserved epitopes would always be presented in a natural protein context.

The Step trial has raised the minimum threshold for the level of T cell immunity that must be elicited in human subjects for an effective outcome. The importance of a high responder frequency and the magnitude, quality, and breadth of response are well appreciated. The result presented here is that both HIV infected subjects and healthy volunteers immunized with the same vaccine as in the Step trial exhibited a response biased towards less-conserved epitopes. Developing an effective T-cell based vaccine as part of an overall HIV-1 vaccine will require eliciting robust responses to well-conserved epitopes. Overcoming this bias will require both an improved understanding of HIV and its interaction with the presentation and processing machinery, probing the underlying causes for potential bias towards less-conserved epitopes, and the refinement of computational design strategies to overcome this bias and refocus the immune response on a broad set of highly conserved epitopes.

## Materials and Methods

### Epitope mapping

Epitope mapping after vaccination was performed on a study cohort based on previously determined positive ELISpot responses to complete 15-mer peptide pools for each protein [31]. We utilized a modified IFN-γ ELISpot assay using peptide minipools matching the vaccine immunogen sequences to determine the breadth of epitope response to the vaccine. Each pool contained eight overlapping 9-amino-acid peptides spanning a 16-amino-acid region. Where sample was sufficient, positive minipool responses were resolved by subsequent ELISpot assay using the individual 9-mer peptides in the responding minipool. Cut-offs to establish a 5% false-positive rate in the individual 9-mer assays were experimentally determined to be ≥4x mock and ≥80 (gag), 55(nef), 70 (pol pool-1) and 80 (pol pool-2) spots/10^6^ PBMC.

### Epitope frequency in circulating strains

130 (one per patient), recently-isolated HIV-1 subtype B genome sequences were obtained from Los Alamos HIV Sequence Database (http://www.hiv.lanl.gov). Intact Gag, Pol and Nef proteins sequences were derived from the corresponding coding regions of the genomes. Frequencies of reactive, known and potential T-cell epitopes were calculated according to their occurrence in the viral sequences. Experimentally defined known HIV CTL epitopes were obtained from the same source.

### Conservation of epitopes relative to clade B circulating strains

Amino acid sequences with unique patient identifiers and designated as subtype B were downloaded from the Los Alamos HIV Sequence Database in August 2009. Due to the design of the Step trial, clade B sequences are the vaccine sequences and expected viral challenge sequences. Sequences were cleaned by removal of gaps and trailing stop codons and replacement of intermediate stop codons and unknown amino acids by a standard character (‘X’). To avoid potential bias due to the submission of partial sequences to the LANL database, only complete or near-complete sequences were included in the analysis. Gag sequences were required to have 500±20 amino acids, Pol 850±20 amino acids, and Nef, 210 −20, 210+14 amino acids. For Gag, 1506 sequences from 461 patients were used; Nef, 5177 sequences from 784 patients; and Pol (RT through IN), 741 sequences from 236 patients.

Sequences were compared as described previously [25]. Briefly, amino acid sequences were compared pairwise by 9-mer amino acid identity, according to two rules. For 9/9: all 9 amino acids much match. For 8/9: 8 out of 9 amino acids much match (the position of mismatch within the 9-mer is irrelevant). Up to one unknown comparison is allowed without penalty. That is, a single X in either N-mer is considered to be identical to the amino acid at the same position in the other N-mer. Calculations were patient-normalized (all patients contribute equally to the analyses, regardless of the number of sequences they contribute to the sequence database).

### Ethics statement

The data presented here are derived from a safety and immunogenicity vaccine study in HIV-seronegative adults [17]. Participants were followed for up to 78 weeks for immunogenicity and 260 weeks for safety. The protocol was approved by review boards at participating centers (V520-016 study group), and written informed consent was obtained from all participants. Ongoing risk assessments and preventive counseling were offered to participants during the trial. Further details on enrollment and exclusion criteria have been previously published [17]. For the current study, participant-specific information was stripped from the data and identifiers were re-randomized before analysis.

## Supporting Information

Figure S1 Comparison of PTE frequency of Los Alamos subtype B Env with those of recently isolated subtype B Env from Chavi study. Frequencies of PTEs are highly correlated (p<0.0001) between the sequence sets.
(TIF)

Table S1 Positive minipools and 9-mer epitopes indentified from Merck Ad5 *gag/pol/nef* vaccine responders
(DOC)

Table S2 Median protein similarity and reactive epitope frequency in subtype B and C strains
(DOC)
