# Supplementary material for: Mapping HIV-1 Vaccine Induced T-Cell Responses: Bias towards Less-Conserved Regions and Potential Impact on Vaccine Efficacy in the Step Study
Source: PLoS One. 2011 Jun 10;6(6):e20479. doi: 10.1371/journal.pone.0020479 (PMC3112144; doi:10.1371/journal.pone.0020479)
Supplement: Table S1 — Positive minipools and 9-mer epitopes indentified from Merck Ad5 gag/pol/nef vaccine responders (DOC) [file pone.0020479.s002.doc]

**Table S1. Positive minipools and 9-mer epitopes indentified from Merck Ad5 *gag/pol/nef* vaccine responders**

| **PID** | **HLA-A** | | **HLA-B** | | **HLA-C** | | **Gene** | **Positive minipool** | **Positive 9-mer** | | |
| --- | --- | --- | --- | --- | --- | --- | --- | --- | --- | --- | --- |
| 1 | A*0205 | A*0301 | B*1501 | B*5001 | CW*0303 | CW*0602 | Gag | EKIRLRPGGKKKYKLK | RLRPGGKKK | LRPGGKKKY |  |
| 2 | A*0201 | A*0301 | B*0801 | B*2705 | CW*0102 | CW*0701 | Gag | EKIRLRPGGKKKYKLK | RLRPGGKKK |  |  |
| 3 | A*0301 | A*2402 | B*1520 | B*0702 | CW*0102 | CW*0702 | Gag | EKIRLRPGGKKKYKLK | RLRPGGKKK |  |  |
| 4 | A*0301 | A*2402 | B*1801 | B*0702 | CW*0702 | CW*1203 | Gag | EKIRLRPGGKKKYKLK | RLRPGGKKK |  |  |
| 5 | A*0201 | A*0301 | B*1302 | B*1501 | CW*0304 | CW*0602 | Gag | EKIRLRPGGKKKYKLK | RLRPGGKKK |  |  |
| 6 | A*0201 | A*0301 | B*0702 | B*3501 | CW*0401 | CW*0702 | Gag | EKIRLRPGGKKKYKLK | RLRPGGKKK |  |  |
| 7 | A*0301 | A*0301 | B*3501 | B*5101 | CW*0401 | CW*1402 | Gag | EKIRLRPGGKKKYKLK | RLRPGGKKK |  |  |
| 8 | A*03XX | A*03XX | B*1402 | B*3501 | CW*0401 | CW*0802 | Gag | EKIRLRPGGKKKYKLK | IRLRPGGKK |  |  |
| 9 | A*0201 | A*0301 | B*0702 | B*4002 | CW*0202 | CW*0702 | Gag | EKIRLRPGGKKKYKLK | not deconv. |  |  |
| 10 | A*0201 | A*0301 | B*0702 | B*3901 | CW*0702 | CW*1203 | Gag | EKIRLRPGGKKKYKLK | not deconv. |  |  |
| 11 | A*0201 | A*0301 | B*0702 | B*4002 | CW*0202 | CW*0702 | Gag | TVATLYCVHQKIDVKD | ATLYCVHQK |  |  |
| 12 | A*0201 | A*1101 | B*4001 | B*4402 | CW*0304 | CW*0501 | Gag | TVATLYCVHQKIDVKD | ATLYCVHQK |  |  |
| 13 | A*1101 | A*3101 | B*1402 | B*4403 | CW*0802 | CW*1601 | Gag | TVATLYCVHQKIDVKD | ATLYCVHQK |  |  |
| 14 | A*0101 | A*1101 | B*1501 | B*4403 | CW*0304 | CW*1601 | Gag | TVATLYCVHQKIDVKD | ATLYCVHQK |  |  |
| 15 | A*0201 | A*1101 | B*0702 | B*5101 | CW*0702 | CW*1502 | Gag | TVATLYCVHQKIDVKD | ATLYCVHQK | VATLYCVHQ |  |
| 16 | A*1101 | A*3002 | B*0702 | B*1401 | CW*0702 | CW*0802 | Gag | TVATLYCVHQKIDVKD | ATLYCVHQK |  |  |
| 17 | A*1101 | A*3001 | B*3501 | B*1302 | CW*0401 | CW*0602 | Gag | TVATLYCVHQKIDVKD | not deconv. |  |  |
| 7 | A*0301 | A*0301 | B*3501 | B*5101 | CW*0401 | CW*1402 | Gag | TVATLYCVHQKIDVKD | not deconv. |  |  |
| 18 | A*0201 | A*2402 | B*1402 | B*3901 | CW*0802 | CW*1203 | Gag | GKKKYKLKHIVWASRE | KYKLKHIVW |  |  |
| 3 | A*0301 | A*2402 | B*1520 | B*0702 | CW*0102 | CW*0702 | Gag | GKKKYKLKHIVWASRE | KYKLKHIVW |  |  |
| 19 | A*2402 | A*3002 | B*0801 | B*1801 | CW*0501 | CW*0701 | Gag | GKKKYKLKHIVWASRE | KYKLKHIVW |  |  |
| 20 | A*2403 | A*6801 | B*3503 | B*3801 | CW*0401 | CW*1203 | Gag | GKKKYKLKHIVWASRE | KYKLKHIVW | YKLKHIVWA |  |
| 10 | A*0201 | A*0301 | B*0702 | B*3901 | CW*0702 | CW*1203 | Gag | GKKKYKLKHIVWASRE | not deconv. |  |  |
| 2 | A*0201 | A*0301 | B*0801 | B*2705 | CW*0102 | CW*0701 | Gag | PVGEIYKRWIILGLNK | RWIILGLNK |  |  |
| 21 | A*0201 | A*0301 | B*0801 | B*2705 | CW*0102 | CW*0701 | Gag | PVGEIYKRWIILGLNK | RWIILGLNK |  |  |
| 22 | A*0201 | A*3303 | B*2705 | B*4402 | CW*0202 | CW*0501 | Gag | PVGEIYKRWIILGLNK | RWIILGLNK |  |  |
| 23 | A*0201 | A*2402 | B*2703 | B*4402 | CW*0102 | CW*0501 | Gag | PVGEIYKRWIILGLNK | RWIILGLNK |  |  |
| 19 | A*2402 | A*3002 | B*0801 | B*1801 | CW*0501 | CW*0701 | Gag | PVGEIYKRWIILGLNK |  |  |  |
| 18 | A*0201 | A*2402 | B*1402 | B*3901 | CW*0802 | CW*1203 | Gag | NLQGQMVHQAISPRTL | HQAISPRTL |  |  |
| 10 | A*0201 | A*0301 | B*0702 | B*3901 | CW*0702 | CW*1203 | Gag | NLQGQMVHQAISPRTL | HQAISPRTL |  |  |
| 24 | A*2402 | A*3001 | B*3901 | B*1302 | CW*0602 | CW*1203 | Gag | NLQGQMVHQAISPRTL | GQMVHQAIS |  |  |
| 7 | A*0301 | A*0301 | B*3501 | B*5101 | CW*0401 | CW*1402 | Gag | NLQGQMVHQAISPRTL |  |  |  |
| 25 | A*0201 | A*0206 | B*5101 |  | CW*0401 | CW*1402 | Gag | NLQGQMVHQAISPRTL | not deconv. |  |  |
| 26 | A*0101 | A*2402 | B*3503 | B*5701 | CW*0401 | CW*0602 | Gag | STLQEQIGWMTNNPPI | STLQEQIGW |  |  |
| 27 | A*2601 | A*2902 | B*5801 | B*5501 | CW*0303 | CW*0701 | Gag | STLQEQIGWMTNNPPI | STLQEQIGW |  |  |
| 28 | A*0201 | A*0205 | B*5101 | B*58XX | CW*0701 | CW*1602 | Gag | STLQEQIGWMTNNPPI | not deconv. |  |  |
| 29 | A*3101 | A*6801 | B*0801 | B*5701 | CW*0304 | CW*0602 | Gag | STLQEQIGWMTNNPPI | not deconv. |  |  |
| 30 | A*0201 | A*0201 | B*4001 | B*4402 | CW*0304 | CW*0501 | Gag | KELYPLASLRSLFGND | KELYPLASL |  |  |
| 31 | A*0201 | A*4301 | B*4001 | B*5301 | CW*0304 | CW*0401 | Gag | KELYPLASLRSLFGND | KELYPLASL |  |  |
| 32 | A*0201 | A*3101 | B*4001 |  | CW*0304 |  | Gag | KELYPLASLRSLFGND | not deconv. |  |  |
| 25 | A*0201 | A*0206 | B*5101 |  | CW*0401 | CW*1402 | Gag | EELRSLYNTVATLYCV | SLYNTVATL | LYNTVATLY |  |
| 7 | A*0301 | A*0301 | B*3501 | B*5101 | CW*0401 | CW*1402 | Gag | EELRSLYNTVATLYCV | SLYNTVATL | LYNTVATLY |  |
| 2 | A*0201 | A*0301 | B*0801 | B*2705 | CW*0102 | CW*0701 | Gag | MMQRGNFRNQRKTVKC | MMQRGNFRN | QRGNFRNQR |  |
| 33 | A*0201 | A*3201 | B*3906 | B*3701 | CW*0602 | CW*0702 | Gag | MMQRGNFRNQRKTVKC | RNQRKTVKC |  |  |
| 34 | A*1101 | A*6801 | B*5301 | B*1518 | CW*0401 | CW*0704 | Gag | QVTNSATIMMQRGNFR | QVTNSATIM | VTNSATIMM |  |
| 20 | A*2403 | A*6801 | B*3503 | B*3801 | CW*0401 | CW*1203 | Gag | QVTNSATIMMQRGNFR | NSATIMMQR |  |  |
| 35 | A*0201 | A*2601 | B*3801 | B*3701 | CW*0602 | CW*1203 | Gag | EKAFSPEVIPMFSALS | EVIPMFSAL |  |  |
| 36 | A*0101 | A*2601 | B*0801 | B*3801 | CW*0701 | CW*1203 | Gag | EKAFSPEVIPMFSALS | EVIPMFSAL |  |  |
| 14 | A*0101 | A*1101 | B*1501 | B*4403 | CW*0304 | CW*1601 | Gag | QPSLQTGSEELRSLYN | GSEELRSLY |  |  |
| 25 | A*0201 | A*0206 | B*5101 |  | CW*0401 | CW*1402 | Gag | QPSLQTGSEELRSLYN | not deconv. |  |  |
| 37 | A*3301 | A*7401 | B*5301 | B*7801 | CW*0401 | CW*1601 | Gag | AAEWDRLHPVHAGPIA | HPVHAGPIA |  |  |
| 7 | A*0301 | A*0301 | B*3501 | B*5101 | CW*0401 | CW*1402 | Gag | AAEWDRLHPVHAGPIA |  |  |  |
| 7 | A*0301 | A*0301 | B*3501 | B*5101 | CW*0401 | CW*1402 | Gag | QAISPRTLNAWVKVVE |  |  |  |
| 11 | A*0201 | A*0301 | B*0702 | B*4002 | CW*0202 | CW*0702 | Gag | QAISPRTLNAWVKVVE | not deconv. |  |  |
| 10 | A*0201 | A*0301 | B*0702 | B*3901 | CW*0702 | CW*1203 | Gag | LERFAVNPGLLETSEG | FAVNPGLLE | NPGLLETSE |  |
| 38 | A*2402 | A*6801 | B*1503 | B*4006 | CW*0202 | CW*1502 | Gag | LKETINEEAAEWDRLH | KETINEEAA |  |  |
| 39 | A*0201 | A*0301 | B*3503 | re pcr | CW*0401 | CW*1203 | Gag | LLVQNANPDCKTILKA | ANPDCKTIL |  |  |
| 37 | A*3301 | A*7401 | B*5301 | B*7801 | CW*0401 | CW*1601 | Gag | RAEQASQEVKNWMTET | QASQEVKNW |  |  |
| 31 | A*0201 | A*4301 | B*4001 | B*5301 | CW*0304 | CW*0401 | Gag | RVLAEAMSQVTNSATI | VLAEAMSQV |  |  |
| 16 | A*1101 | A*3002 | B*0702 | B*1401 | CW*0702 | CW*0802 | Gag | VDRFYKTLRAEQASQE | DRFYKTLRA |  |  |
| 19 | A*2402 | A*3002 | B*0801 | B*1801 | CW*0501 | CW*0701 | Gag | HQKIDVKDTKEALEKI |  |  |  |
| 10 | A*0201 | A*0301 | B*0702 | B*3901 | CW*0702 | CW*1203 | Gag | HIVWASRELERFAVNP | not deconv. |  |  |
| 23 | A*0201 |  |  |  |  |  | Gag | LGPAATLEEMMTACQG | not deconv. |  |  |
| 10 | A*0201 | A*0301 | B*0702 | B*3901 | CW*0702 | CW*1203 | Gag | PVHAGPIAPGQMREPR | not deconv. |  |  |
| 25 | A*0201 | A*0206 | B*5101 |  | CW*0401 | CW*1402 | Gag | SQNYPIVQNLQGQMVH | not deconv. |  |  |
| 11 | A*0201 | A*0301 | B*0702 | B*4002 | CW*0202 | CW*0702 | Gag | VGGPGHKARVLAEAMS | not deconv. |  |  |
| 18 | A*0201 | A*2402 | B*1402 | B*3901 | CW*0802 | CW*1203 | Nef | PAADRVRRTEPAAVGV | DRVRRTEPA |  |  |
| 40 | A*0201 |  | B*4402 | B*1402 | CW*0501 | CW*0802 | Nef | PAADRVRRTEPAAVGV | DRVRRTEPA |  |  |
| 24 | A*2402 | A*3001 | B*3901 | B*1302 | CW*0602 | CW*1203 | Nef | PAADRVRRTEPAAVGV | RVRRTEPAA |  |  |
| 3 | A*0301 | A*2402 | B*1520 | B*0702 | CW*0102 | CW*0702 | Nef | PAADRVRRTEPAAVGV | RVRRTEPAA |  |  |
| 41 | A*0101 | A*0201 | B*1302 | B*5701 | CW*0602 |  | Nef | PAADRVRRTEPAAVGV | RVRRTEPAA |  |  |
| 42 | A*0201 |  | B*0702 | B*3503 | CW*0702 | CW*1203 | Nef | PAADRVRRTEPAAVGV | RVRRTEPAA | VRRTEPAAV |  |
| 5 | A*0201 | A*0301 | B*1302 | B*1501 | CW*0304 | CW*0602 | Nef | PAADRVRRTEPAAVGV | RVRRTEPAA |  |  |
| 10 | A*0201 | A*0301 | B*0702 | B*3901 | CW*0702 | CW*1203 | Nef | PAADRVRRTEPAAVGV | not deconv. |  |  |
| 11 | A*0201 | A*0301 | B*0702 | B*4002 | CW*0202 | CW*0702 | Nef | MTYKGAVDLSHFLKEK | AVDLSHFLK |  |  |
| 13 | A*1101 | A*3101 | B*1402 | B*4403 | CW*0802 | CW*1601 | Nef | MTYKGAVDLSHFLKEK | AVDLSHFLK |  |  |
| 14 | A*0101 | A*1101 | B*1501 | B*4403 | CW*0304 | CW*1601 | Nef | MTYKGAVDLSHFLKEK | AVDLSHFLK |  |  |
| 15 | A*0201 | A*1101 | B*0702 | B*5101 | CW*0702 | CW*1502 | Nef | MTYKGAVDLSHFLKEK | AVDLSHFLK |  |  |
| 16 | A*1101 | A*3002 | B*0702 | B*1401 | CW*0702 | CW*0802 | Nef | MTYKGAVDLSHFLKEK | AVDLSHFLK |  |  |
| 1 | A*0205 | A*0301 | B*1501 | B*5001 | CW*0303 | CW*0602 | Nef | MTYKGAVDLSHFLKEK | GAVDLSHFL |  |  |
| 28 | A*0201 | A*0205 | B*5101 | B*58XX | CW*0701 | CW*1602 | Nef | MTYKGAVDLSHFLKEK | not deconv. |  |  |
| 17 | A*1101 | A*3001 | B*3501 | B*1302 | CW*0401 | CW*0602 | Nef | MTYKGAVDLSHFLKEK | not deconv. |  |  |
| 2 | A*0201 | A*0301 | B*0801 | B*2705 | CW*0102 | CW*0701 | Nef | WRFDSKLAFHHVAREL | WRFDSKLAF |  |  |
| 21 | A*0201 | A*0301 | B*0801 | B*2705 | CW*0102 | CW*0701 | Nef | WRFDSKLAFHHVAREL | WRFDSKLAF |  |  |
| 22 | A*0201 | A*3303 | B*2705 | B*4402 | CW*0202 | CW*0501 | Nef | WRFDSKLAFHHVAREL | WRFDSKLAF |  |  |
| 37 | A*3301 | A*7401 | B*5301 | B*7801 | CW*0401 | CW*1601 | Nef | WRFDSKLAFHHVAREL | KLAFHHVAR |  |  |
| 7 | A*0301 | A*0301 | B*3501 | B*5101 | CW*0401 | CW*1402 | Nef | WRFDSKLAFHHVAREL | AFHHVAREL |  |  |
| 43 | A*0101 | A*0201 | B*1401 | B*4001 | CW*0304 | CW*0802 | Nef | PMSQHGIEDPEKEVLE | IEDPEKEVL |  |  |
| 32 | A*0201 | A*3101 | B*4001 |  | CW*0304 |  | Nef | PMSQHGIEDPEKEVLE | IEDPEKEVL |  |  |
| 30 | A*0201 | A*0201 | B*4001 | B*4402 | CW*0304 | CW*0501 | Nef | PMSQHGIEDPEKEVLE | IEDPEKEVL |  |  |
| 31 | A*0201 | A*4301 | B*4001 | B*5301 | CW*0304 | CW*0401 | Nef | PMSQHGIEDPEKEVLE | IEDPEKEVL |  |  |
| 44 | A*0201 | A*1101 | B*1301 | B*4001 | CW*0304 | CW*0702 | Nef | PMSQHGIEDPEKEVLE | not deconv. |  |  |
| 14 | A*0101 | A*1101 | B*1501 | B*4403 | CW*0304 | CW*1601 | Nef | DEEVGFPVRPQVPLRP | VGFPVRPQV |  |  |
| 15 | A*0201 | A*1101 | B*0702 | B*5101 | CW*0702 | CW*1502 | Nef | DEEVGFPVRPQVPLRP | VGFPVRPQV |  |  |
| 11 | A*0201 | A*0301 | B*0702 | B*4002 | CW*0202 | CW*0702 | Nef | DEEVGFPVRPQVPLRP |  |  |  |
| 26 | A*0101 | A*2402 | B*3503 | B*5701 | CW*0401 | CW*0602 | Nef | DEEVGFPVRPQVPLRP |  |  |  |
| 16 | A*1101 | A*3002 | B*0702 | B*1401 | CW*0702 | CW*0802 | Nef | DEEVGFPVRPQVPLRP | not deconv. |  |  |
| 45 | A*0201 |  | B*5801 | B*1501 | CW*0302 | CW*0303 | Nef | NTAATNADCAWLEAQE | AATNADCAW |  |  |
| 26 | A*0101 | A*2402 | B*3503 | B*5701 | CW*0401 | CW*0602 | Nef | NTAATNADCAWLEAQE | AATNADCAW |  |  |
| 27 | A*2601 | A*2902 | B*5801 | B*5501 | CW*0303 | CW*0701 | Nef | NTAATNADCAWLEAQE | AATNADCAW |  |  |
| 37 | A*3301 | A*7401 | B*5301 | B*7801 | CW*0401 | CW*1601 | Nef | NTAATNADCAWLEAQE | AATNADCAW |  |  |
| 46 | A*0201 | A*2601 | B*4402 | B*5701 | CW*0501 | CW*0602 | Nef | MGGKWSKRSVPGWSTV | WSKRSVPGW |  |  |
| 47 | A*0101 | A*0201 | B*1801 | B*5701 | CW*0602 | CW*0701 | Nef | MGGKWSKRSVPGWSTV | WSKRSVPGW |  |  |
| 26 | A*0101 | A*2402 | B*3503 | B*5701 | CW*0401 | CW*0602 | Nef | MGGKWSKRSVPGWSTV | WSKRSVPGW | SKRSVPGWS | |
| 24 | A*2402 | A*3001 | B*3901 | B*1302 | CW*0602 | CW*1203 | Nef | RERMRRAEPAADRVRR | RMRRAEPAA |  |  |
| 3 | A*0301 | A*2402 | B*1520 | B*0702 | CW*0102 | CW*0702 | Nef | RERMRRAEPAADRVRR | RMRRAEPAA |  |  |
| 5 | A*0201 | A*0301 | B*1302 | B*1501 | CW*0304 | CW*0602 | Nef | RERMRRAEPAADRVRR | RMRRAEPAA |  |  |
| 35 | A*0201 | A*2601 | B*3801 | B*3701 | CW*0602 | CW*1203 | Nef | GGLEGLIHSQKRQDIL | HSQKRQDIL |  |  |
| 36 | A*0101 | A*2601 | B*0801 | B*3801 | CW*0701 | CW*1203 | Nef | GGLEGLIHSQKRQDIL | not deconv. |  |  |
| 10 | A*0201 | A*0301 | B*0702 | B*3901 | CW*0702 | CW*1203 | Nef | GGLEGLIHSQKRQDIL | not deconv. |  |  |
| 33 | A*0201 | A*3201 | B*3906 | B*3701 | CW*0602 | CW*0702 | Nef | GAVSRDLEKHGAITSS | RDLEKHGAI |  |  |
| 35 | A*0201 | A*2601 | B*3801 | B*3701 | CW*0602 | CW*1203 | Nef | GAVSRDLEKHGAITSS | RDLEKHGAI |  |  |
| 48 | A*0101 | A*3201 | B*0801 |  | CW*0701 |  |  | LSHFLKEKGGLEGLIH | HFLKEKGGL |  |  |
| 19 | A*2402 | A*3002 | B*0801 | B*1801 | CW*0501 | CW*0701 | Nef | LSHFLKEKGGLEGLIH | HFLKEKGGL |  |  |
| 26 | A*0101 | A*2402 | B*3503 | B*5701 | CW*0401 | CW*0602 | Nef | DLWVYHTQGYFPDWQN | HTQGYFPDW |  |  |
| 10 | A*0201 | A*0301 | B*0702 | B*3901 | CW*0702 | CW*1203 | Nef | DLWVYHTQGYFPDWQN | not deconv. |  |  |
| 36 | A*0101 | A*2601 | B*0801 | B*3801 | CW*0701 | CW*1203 | Nef | GENNCLLHPMSQHGIE | not deconv. |  |  |
| 10 | A*0201 | A*0301 | B*0702 | B*3901 | CW*0702 | CW*1203 | Nef | GENNCLLHPMSQHGIE | not deconv. |  |  |
| 37 | A*3301 | A*7401 | B*5301 | B*7801 | CW*0401 | CW*1601 | Nef | DPEKEVLEWRFDSKLA | DPEKEVLEW |  |  |
| 20 | A*2403 | A*6801 | B*3503 | B*3801 | CW*0401 | CW*1203 | Nef | SVPGWSTVRERMRRAE | SVPGWSTVR |  |  |
| 11 | A*0201 | A*0301 | B*0702 | B*4002 | CW*0202 | CW*0702 | Nef | CAWLEAQEDEEVGFPV | not deconv. |  |  |
| 39 | A*0201 | A*0301 | B*3503 | re pcr | CW*0401 | CW*1203 | Nef | FHHVARELHPEYYKDC | not deconv. |  |  |
| 18 | A*0201 | A*2402 | B*1402 | B*3901 | CW*0802 | CW*1203 | Pol | ITTESIVIWGKTPKFK | ITTESIVIW |  |  |
| 46 | A*0201 | A*2601 | B*4402 | B*5701 | CW*0501 | CW*0602 | Pol | ITTESIVIWGKTPKFK | ITTESIVIW |  |  |
| 27 | A*2601 | A*2902 | B*5801 | B*5501 | CW*0303 | CW*0701 | Pol | ITTESIVIWGKTPKFK | ITTESIVIW |  |  |
| 4 | A*0301 | A*2402 | B*1801 | B*0702 | CW*0702 | CW*1203 | Pol | ITTESIVIWGKTPKFK | ITTESIVIW |  |  |
| 36 | A*0101 | A*2601 | B*0801 | B*3801 | CW*0701 | CW*1203 | Pol | ITTESIVIWGKTPKFK | ITTESIVIW |  |  |
| 26 | A*0101 | A*2402 | B*3503 | B*5701 | CW*0401 | CW*0602 | Pol | ITTESIVIWGKTPKFK |  |  |  |
| 21 | A*0201 | A*0301 | B*0801 | B*2705 | CW*0102 | CW*0701 | Pol | QGWKGSPAIFQSSMTK | AIFQSSMTK |  |  |
| 14 | A*0101 | A*1101 | B*1501 | B*4403 | CW*0304 | CW*1601 | Pol | QGWKGSPAIFQSSMTK | AIFQSSMTK | GSPAIFQSS | QGWKGSPAI |
| 4 | A*0301 | A*2402 | B*1801 | B*0702 | CW*0702 | CW*1203 | Pol | QGWKGSPAIFQSSMTK | AIFQSSMTK | SPAIFQSSM |  |
| 49 | A*2301 | A*3402 | B*0702 |  | CW*0702 |  | Pol | QGWKGSPAIFQSSMTK | SPAIFQSSM |  |  |
| 44 | A*0201 | A*1101 | B*1301 | B*4001 | CW*0304 | CW*0702 | Pol | QGWKGSPAIFQSSMTK | not deconv. |  |  |
| 13 | A*1101 | A*3101 | B*1402 | B*4403 | CW*0802 | CW*1601 | Pol | QGWKGSPAIFQSSMTK | not deconv. |  |  |
| 50 | A*0201 | A*3002 | B*3801 | B*3901 | CW*1203 | CW*1701 | Pol | KIQNFRVYYRDSRNPL | KIQNFRVYY |  |  |
| 33 | A*0201 | A*3201 | B*3906 | B*3701 | CW*0602 | CW*0702 | Pol | KIQNFRVYYRDSRNPL | KIQNFRVYY |  |  |
| 51 | A*2501 | A*3002 | B*1801 | B*4402 | CW*0501 | CW*0501 | Pol | KIQNFRVYYRDSRNPL | KIQNFRVYY | IQNFRVYYR |  |
| 20 | A*2403 | A*6801 | B*3503 | B*3801 | CW*0401 | CW*1203 | Pol | KIQNFRVYYRDSRNPL |  |  |  |
| 37 | A*3301 | A*7401 | B*5301 | B*7801 | CW*0401 | CW*1601 | Pol | KIQNFRVYYRDSRNPL | not deconv. |  |  |
| 19 | A*2402 | A*3002 | B*0801 | B*1801 | CW*0501 | CW*0701 | Pol | KQITKIQNFRVYYRDS | KIQNFRVYY |  |  |
| 16 | A*1101 | A*3002 | B*0702 | B*1401 | CW*0702 | CW*0802 | Pol | KQITKIQNFRVYYRDS | KIQNFRVYY |  |  |
| 52 | A*0207 | A*2402 | B*0702 | B*5101 | CW*0702 |  | Pol | NLPPVVAKEIVASCDK | LPPVVAKEI |  |  |
| 15 | A*0201 | A*1101 | B*0702 | B*5101 | CW*0702 | CW*1502 | Pol | NLPPVVAKEIVASCDK | LPPVVAKEI |  |  |
| 7 | A*0301 | A*0301 | B*3501 | B*5101 | CW*0401 | CW*1402 | Pol | NLPPVVAKEIVASCDK | LPPVVAKEI |  |  |
| 20 | A*2403 | A*6801 | B*3503 | B*3801 | CW*0401 | CW*1203 | Pol | NLPPVVAKEIVASCDK |  |  |  |
| 53 | A*2902 | A*2902 | B*0702 | B*3503 | CW*0401 | CW*0702 | Pol | NLPPVVAKEIVASCDK | not deconv. |  |  |
| 46 | A*0201 | A*2601 | B*4402 | B*5701 | CW*0501 | CW*0602 | Pol | WTVQPIVLPEKDSWTV | IVLPEKDSW |  |  |
| 41 | A*0101 | A*0201 | B*1302 | B*5701 | CW*0602 |  | Pol | WTVQPIVLPEKDSWTV | IVLPEKDSW |  |  |
| 54 | A*0101 | A*3303 | B*2702 | B*5701 | CW*0202 | CW*0602 | Pol | WTVQPIVLPEKDSWTV | IVLPEKDSW |  |  |
| 26 | A*0101 | A*2402 | B*3503 | B*5701 | CW*0401 | CW*0602 | Pol | WTVQPIVLPEKDSWTV | IVLPEKDSW | TVQPIVLPE |  |
| 24 | A*2402 | A*3001 | B*3901 | B*1302 | CW*0602 | CW*1203 | Pol | QLTEAVQKITTESIVI | VQKITTESI | QLTEAVQKI |  |
| 5 | A*0201 | A*0301 | B*1302 | B*1501 | CW*0304 | CW*0602 | Pol | QLTEAVQKITTESIVI | VQKITTESI | QLTEAVQKI |  |
| 41 | A*0101 | A*0201 | B*1302 | B*5701 | CW*0602 |  | Pol | QLTEAVQKITTESIVI | VQKITTESI |  |  |
| 17 | A*1101 | A*3001 | B*3501 | B*1302 | CW*0401 | CW*0602 | Pol | QLTEAVQKITTESIVI | not deconv. |  |  |
| 18 | A*0201 | A*2402 | B*1402 | B*3901 | CW*0802 | CW*1203 | Pol | YTAFTIPSINNETPGI | YTAFTIPSI |  |  |
| 2 | A*0201 | A*0301 | B*0801 | B*2705 | CW*0102 | CW*0701 | Pol | YTAFTIPSINNETPGI | YTAFTIPSI |  |  |
| 7 | A*0301 | A*0301 | B*3501 | B*5101 | CW*0401 | CW*1402 | Pol | YTAFTIPSINNETPGI | YTAFTIPSI | TAFTIPSIN |  |
| 55 | A*0201 | A*2403 | B*1801 | B*4402 | CW*0501 | CW*1203 | Pol | YTAFTIPSINNETPGI | not deconv. |  |  |
| 42 | A*0201 |  | B*0702 | B*3503 | CW*0702 | CW*1203 | Pol | ILEPFRKQNPDIVIYQ | QNPDIVIYQ | FRKQNPDIV |  |
| 14 | A*0101 | A*1101 | B*1501 | B*4403 | CW*0304 | CW*1601 | Pol | ILEPFRKQNPDIVIYQ | KQNPDIVIY |  |  |
| 38 | A*2402 | A*6801 | B*1503 | B*4006 | CW*0202 | CW*1502 | Pol | ILEPFRKQNPDIVIYQ | KQNPDIVIY |  |  |
| 3 | A*0301 | A*2402 | B*1520 | B*0702 | CW*0102 | CW*0702 | Pol | ILEPFRKQNPDIVIYQ | not deconv. |  |  |
| 2 | A*0201 | A*0301 | B*0801 | B*2705 | CW*0102 | CW*0701 | Pol | AVFIHNFKRKGGIGGY | KRKGGIGGY |  |  |
| 21 | A*0201 | A*0301 | B*0801 | B*2705 | CW*0102 | CW*0701 | Pol | AVFIHNFKRKGGIGGY | KRKGGIGGY |  |  |
| 22 | A*0201 | A*3303 | B*2705 | B*4402 | CW*0202 | CW*0501 | Pol | AVFIHNFKRKGGIGGY | KRKGGIGGY |  |  |
| 37 | A*3301 | A*7401 | B*5301 | B*7801 | CW*0401 | CW*1601 | Pol | AVFIHNFKRKGGIGGY | not deconv. |  |  |
| 7 | A*0301 | A*0301 | B*3501 | B*5101 | CW*0401 | CW*1402 | Pol | NPDIVIYQYMDDLYVG | NPDIVIYQY |  |  |
| 8 | A*03XX | A*03XX | B*1402 | B*3501 | CW*0401 | CW*0802 | Pol | NPDIVIYQYMDDLYVG | NPDIVIYQY |  |  |
| 26 | A*0101 | A*2402 | B*3503 | B*5701 | CW*0401 | CW*0602 | Pol | NPDIVIYQYMDDLYVG |  |  |  |
| 20 | A*2403 | A*6801 | B*3503 | B*3801 | CW*0401 | CW*1203 | Pol | NPDIVIYQYMDDLYVG |  |  |  |
| 37 | A*3301 | A*7401 | B*5301 | B*7801 | CW*0401 | CW*1601 | Pol | YQLEKEPIVGAETFYV | EPIVGAETF |  |  |
| 20 | A*2403 | A*6801 | B*3503 | B*3801 | CW*0401 | CW*1203 | Pol | YQLEKEPIVGAETFYV | PIVGAETFY |  |  |
| 7 | A*0301 | A*0301 | B*3501 | B*5101 | CW*0401 | CW*1402 | Pol | YQLEKEPIVGAETFYV | not deconv. |  |  |
| 53 | A*2902 | A*2902 | B*0702 | B*3503 | CW*0401 | CW*0702 | Pol | YQLEKEPIVGAETFYV | not deconv. |  |  |
| 2 | A*0201 | A*0301 | B*0801 | B*2705 | CW*0102 | CW*0701 | Pol | RWPVKTIHTDNGSNFT | IHTDNGSNF |  |  |
| 20 | A*2403 | A*6801 | B*3503 | B*3801 | CW*0401 | CW*1203 | Pol | RWPVKTIHTDNGSNFT |  |  |  |
| 17 | A*1101 | A*3001 | B*3501 | B*1302 | CW*0401 | CW*0602 | Pol | RWPVKTIHTDNGSNFT | not deconv. |  |  |
| 53 | A*2902 | A*2902 | B*0702 | B*3503 | CW*0401 | CW*0702 | Pol | RWPVKTIHTDNGSNFT | not deconv. |  |  |
| 20 | A*2403 | A*6801 | B*3503 | B*3801 | CW*0401 | CW*1203 | Pol | VAVHVASGYIEAEVIP |  |  |  |
| 53 | A*2902 | A*2902 | B*0702 | B*3503 | CW*0401 | CW*0702 | Pol | VAVHVASGYIEAEVIP |  |  |  |
| 37 | A*3301 | A*7401 | B*5301 | B*7801 | CW*0401 | CW*1601 | Pol | VAVHVASGYIEAEVIP | not deconv. |  |  |
| 30 | A*0201 | A*0201 | B*4001 | B*4402 | CW*0304 | CW*0501 | Pol | VAVHVASGYIEAEVIP | not deconv. |  |  |
| 37 | A*3301 | A*7401 | B*5301 | B*7801 | CW*0401 | CW*1601 | Pol | LEVNIVTDSQYALGII |  |  |  |
| 20 | A*2403 | A*6801 | B*3503 | B*3801 | CW*0401 | CW*1203 | Pol | LEVNIVTDSQYALGII |  |  |  |
| 7 | A*0301 | A*0301 | B*3501 | B*5101 | CW*0401 | CW*1402 | Pol | LEVNIVTDSQYALGII | not deconv. |  |  |
| 53 | A*2902 | A*2902 | B*0702 | B*3503 | CW*0401 | CW*0702 | Pol | LEVNIVTDSQYALGII | not deconv. |  |  |
| 43 | A*0101 | A*0201 | B*1401 | B*4001 | CW*0304 | CW*0802 | Pol | RTKIEELRQHLLRWGL | IEELRQHLL |  |  |
| 31 | A*0201 | A*4301 | B*4001 | B*5301 | CW*0304 | CW*0401 | Pol | RTKIEELRQHLLRWGL | IEELRQHLL |  |  |
| 14 | A*0101 | A*1101 | B*1501 | B*4403 | CW*0304 | CW*1601 | Pol | RTKIEELRQHLLRWGL | ELRQHLLRW | RQHLLRWGL |  |
| 56 | A*1101 | A*1101 | B*3501 | B*4402 | CW*0401 | CW*0704 | Pol | PLDEDFRKYTAFTIPS | PLDEDFRKY |  |  |
| 7 | A*0301 | A*0301 | B*3501 | B*5101 | CW*0401 | CW*1402 | Pol | PLDEDFRKYTAFTIPS | PLDEDFRKY |  |  |
| 8 | A*03XX | A*03XX | B*1402 | B*3501 | CW*0401 | CW*0802 | Pol | PLDEDFRKYTAFTIPS | PLDEDFRKY |  |  |
| 20 | A*2403 | A*6801 | B*3503 | B*3801 | CW*0401 | CW*1203 | Pol | YLALQDSGLEVNIVTD | GLEVNIVTD | LALQDSGLE | SGLEVNIVT |
| 53 | A*2902 | A*2902 | B*0702 | B*3503 | CW*0401 | CW*0702 | Pol | YLALQDSGLEVNIVTD | GLEVNIVTD | LALQDSGLE |  |
| 26 | A*0101 | A*2402 | B*3503 | B*5701 | CW*0401 | CW*0602 | Pol | QDSGLEVNIVTDSQYA | GLEVNIVTD |  |  |
| 20 | A*2403 | A*6801 | B*3503 | B*3801 | CW*0401 | CW*1203 | Pol | SELVNQIIEQLIKKEK | NQIIEQLIK | IIEQLIKKE |  |
| 53 | A*2902 | A*2902 | B*0702 | B*3503 | CW*0401 | CW*0702 | Pol | SELVNQIIEQLIKKEK | NQIIEQLIK |  |  |
| 26 | A*0101 | A*2402 | B*3503 | B*5701 | CW*0401 | CW*0602 | Pol | NQIIEQLIKKEKVYLA | NQIIEQLIK | IKKEKVYLA |  |
| 43 | A*0101 | A*0201 | B*1401 | B*4001 | CW*0304 | CW*0802 | Pol | MAPISPIETVPVKLKP | PIETVPVKL | IETVPVKLK |  |
| 31 | A*0201 | A*4301 | B*4001 | B*5301 | CW*0304 | CW*0401 | Pol | MAPISPIETVPVKLKP | PIETVPVKL | IETVPVKLK |  |
| 44 | A*0201 | A*1101 | B*1301 | B*4001 | CW*0304 | CW*0702 | Pol | MAPISPIETVPVKLKP | not deconv. |  |  |
| 14 | A*0101 | A*1101 | B*1501 | B*4403 | CW*0304 | CW*1601 | Pol | LPIQKETWETWWTEYW | WETWWTEYW | |  |
| 30 | A*0201 | A*0201 | B*4001 | B*4402 | CW*0304 | CW*0501 | Pol | LPIQKETWETWWTEYW | WETWWTEYW | |  |
| 26 | A*0101 | A*2402 | B*3503 | B*5701 | CW*0401 | CW*0602 | Pol | LPIQKETWETWWTEYW |  |  |  |
| 26 | A*0101 | A*2402 | B*3503 | B*5701 | CW*0401 | CW*0602 | Pol | YMDDLYVGSDLEIGQH | DLYVGSDLE |  |  |
| 20 | A*2403 | A*6801 | B*3503 | B*3801 | CW*0401 | CW*1203 | Pol | YMDDLYVGSDLEIGQH | DLYVGSDLE |  |  |
| 42 | A*0201 |  | B*0702 | B*3503 | CW*0702 | CW*1203 | Pol | YMDDLYVGSDLEIGQH | not deconv. |  |  |
| 20 | A*2403 | A*6801 | B*3503 | B*3801 | CW*0401 | CW*1203 | Pol | TDNGSNFTGATVRAAC | SNFTGATVR |  |  |
| 34 | A*1101 | A*6801 | B*5301 | B*1518 | CW*0401 | CW*0704 | Pol | SNFTGATVRAACWWAG | SNFTGATVR |  |  |
| 26 | A*0101 | A*2402 | B*3503 | B*5701 | CW*0401 | CW*0602 | Pol | SNFTGATVRAACWWAG |  |  |  |
| 8 | A*03XX | A*03XX | B*1402 | B*3501 | CW*0401 | CW*0802 | Pol | YPGIKVRQLCKLLRGT | PGIKVRQLC |  |  |
| 18 | A*0201 | A*2402 | B*1402 | B*3901 | CW*0802 | CW*1203 | Pol | YPGIKVRQLCKLLRGT |  |  |  |
| 7 | A*0301 | A*0301 | B*3501 | B*5101 | CW*0401 | CW*1402 | Pol | YPGIKVRQLCKLLRGT |  |  |  |
| 36 | A*0101 | A*2601 | B*0801 | B*3801 | CW*0701 | CW*1203 | Pol | EHEKYHSNWRAMASDF | EHEKYHSNW |  |  |
| 20 | A*2403 | A*6801 | B*3503 | B*3801 | CW*0401 | CW*1203 | Pol | EHEKYHSNWRAMASDF |  |  |  |
| 57 | A*0101 | A*2902 | B*1402 | B*3801 | CW*0802 | CW*1203 | Pol | EHEKYHSNWRAMASDF | not deconv. |  |  |
| 8 | A*03XX | A*03XX | B*1402 | B*3501 | CW*0401 | CW*0802 | Pol | LAENREILKEPVHGVY | AENREILKE |  |  |
| 7 | A*0301 | A*0301 | B*3501 | B*5101 | CW*0401 | CW*1402 | Pol | LAENREILKEPVHGVY |  |  |  |
| 6 | A*0201 | A*0301 | B*0702 | B*3501 | CW*0401 | CW*0702 | Pol | LAENREILKEPVHGVY | not deconv. |  |  |
| 26 | A*0101 | A*2402 | B*3503 | B*5701 | CW*0401 | CW*0602 | Pol | WGKTPKFKLPIQKETW |  |  |  |
| 20 | A*2403 | A*6801 | B*3503 | B*3801 | CW*0401 | CW*1203 | Pol | WGKTPKFKLPIQKETW |  |  |  |
| 44 | A*0201 | A*1101 | B*1301 | B*4001 | CW*0304 | CW*0702 | Pol | WGKTPKFKLPIQKETW | not deconv. |  |  |
| 26 | A*0101 | A*2402 | B*3503 | B*5701 | CW*0401 | CW*0602 | Pol | KALTEVIPLTEEAELE | VIPLTEEAE | IPLTEEAEL |  |
| 20 | A*2403 | A*6801 | B*3503 | B*3801 | CW*0401 | CW*1203 | Pol | KALTEVIPLTEEAELE | VIPLTEEAE | IPLTEEAEL |  |
| 3 | A*0301 | A*2402 | B*1520 | B*0702 | CW*0102 | CW*0702 | Pol | AEIQKQGQGQWTYQIY | KQGQGQWTY |  |  |
| 14 | A*0101 | A*1101 | B*1501 | B*4403 | CW*0304 | CW*1601 | Pol | AEIQKQGQGQWTYQIY | KQGQGQWTY |  |  |
| 7 | A*0301 | A*0301 | B*3501 | B*5101 | CW*0401 | CW*1402 | Pol | IFQSSMTKILEPFRKQ | SMTKILEPF |  |  |
| 8 | A*03XX | A*03XX | B*1402 | B*3501 | CW*0401 | CW*0802 | Pol | IFQSSMTKILEPFRKQ | SMTKILEPF |  |  |
| 20 | A*2403 | A*6801 | B*3503 | B*3801 | CW*0401 | CW*1203 | Pol | FGIPYNPQSQGVVESM | GIPYNPQSQ |  |  |
| 34 | A*1101 | A*6801 | B*5301 | B*1518 | CW*0401 | CW*0704 | Pol | IKQEFGIPYNPQSQGV | GIPYNPQSQ |  |  |
| 25 | A*0201 | A*0206 | B*5101 |  | CW*0401 | CW*1402 | Pol | LQAIYLALQDSGLEVN | LQAIYLALQ |  |  |
| 26 | A*0101 | A*2402 | B*3503 | B*5701 | CW*0401 | CW*0602 | Pol | LQAIYLALQDSGLEVN |  |  |  |
| 38 | A*2402 | A*6801 | B*1503 | B*4006 | CW*0202 | CW*1502 | Pol | SAGERIVDIIATDIQT | GERIVDIIA |  |  |
| 53 | A*2902 | A*2902 | B*0702 | B*3503 | CW*0401 | CW*0702 | Pol | SAGERIVDIIATDIQT |  |  |  |
| 37 | A*3301 | A*7401 | B*5301 | B*7801 | CW*0401 | CW*1601 | Pol | VYLAWVPAHKGIGGNE | YLAWVPAHK |  |  |
| 20 | A*2403 | A*6801 | B*3503 | B*3801 | CW*0401 | CW*1203 | Pol | VYLAWVPAHKGIGGNE |  |  |  |
| 8 | A*03XX | A*03XX | B*1402 | B*3501 | CW*0401 | CW*0802 | Pol | GATVRAACWWAGIKQE | GATVRAACW |  |  |
| 7 | A*0301 | A*0301 | B*3501 | B*5101 | CW*0401 | CW*1402 | Pol | GATVRAACWWAGIKQE |  |  |  |
| 24 | A*2402 | A*3001 | B*3901 | B*1302 | CW*0602 | CW*1203 | Pol | GIWQLDCTHLEGKVIL | THLEGKVIL |  |  |
| 18 | A*0201 | A*2402 | B*1402 | B*3901 | CW*0802 | CW*1203 | Pol | GIWQLDCTHLEGKVIL |  |  |  |
| 19 | A*2402 | A*3002 | B*0801 | B*1801 | CW*0501 | CW*0701 | Pol | GEAMHGQVDCSPGIWQ |  |  |  |
| 16 | A*1101 | A*3002 | B*0702 | B*1401 | CW*0702 | CW*0802 | Pol | GEAMHGQVDCSPGIWQ |  |  |  |
| 19 | A*2402 | A*3002 | B*0801 | B*1801 | CW*0501 | CW*0701 | Pol | IVTDSQYALGIIQAQP |  |  |  |
| 16 | A*1101 | A*3002 | B*0702 | B*1401 | CW*0702 | CW*0802 | Pol | IVTDSQYALGIIQAQP |  |  |  |
| 7 | A*0301 | A*0301 | B*3501 | B*5101 | CW*0401 | CW*1402 | Pol | FWEVQLGIPHPAGLKK |  |  |  |
| 42 | A*0201 |  | B*0702 | B*3503 | CW*0702 | CW*1203 | Pol | FWEVQLGIPHPAGLKK | not deconv. |  |  |
| 53 | A*2902 | A*2902 | B*0702 | B*3503 | CW*0401 | CW*0702 | Pol | KELQKQITKIQNFRVY |  |  |  |
| 37 | A*3301 | A*7401 | B*5301 | B*7801 | CW*0401 | CW*1601 | Pol | KELQKQITKIQNFRVY | not deconv. |  |  |
| 20 | A*2403 | A*6801 | B*3503 | B*3801 | CW*0401 | CW*1203 | Pol | QWPLTEEKIKALVEIC |  |  |  |
| 7 | A*0301 | A*0301 | B*3501 | B*5101 | CW*0401 | CW*1402 | Pol | QWPLTEEKIKALVEIC |  |  |  |
| 7 | A*0301 | A*0301 | B*3501 | B*5101 | CW*0401 | CW*1402 | Pol | RKGGIGGYSAGERIVD |  |  |  |
| 6 | A*0201 | A*0301 | B*0702 | B*3501 | CW*0401 | CW*0702 | Pol | RKGGIGGYSAGERIVD | not deconv. |  |  |
| 18 | A*0201 | A*2402 | B*1402 | B*3901 | CW*0802 | CW*1203 | Pol | SDLEIGQHRTKIEELR |  |  |  |
| 37 | A*3301 | A*7401 | B*5301 | B*7801 | CW*0401 | CW*1601 | Pol | SDLEIGQHRTKIEELR | not deconv. |  |  |
| 7 | A*0301 | A*0301 | B*3501 | B*5101 | CW*0401 | CW*1402 | Pol | TTPDKKHQKEPPFLWM |  |  |  |
| 14 | A*0101 | A*1101 | B*1501 | B*4403 | CW*0304 | CW*1601 | Pol | TTPDKKHQKEPPFLWM | not deconv. |  |  |
| 2 | A*0201 | A*0301 | B*0801 | B*2705 | CW*0102 | CW*0701 | Pol | YFLLKLAGRWPVKTIH | not deconv. |  |  |
| 4 | A*0301 | A*2402 | B*1801 | B*0702 | CW*0702 | CW*1203 | Pol | YFLLKLAGRWPVKTIH | not deconv. |  |  |
| 42 | A*0201 |  | B*0702 | B*3503 | CW*0702 | CW*1203 | Pol | KKSVTVLDVGDAYFSV | SVTVLDVGD |  |  |
| 55 | A*0201 | A*2403 | B*1801 | B*4402 | CW*0501 | CW*1203 | Pol | INNETPGIRYQYNVLP | NETPGIRYQ |  |  |
| 36 | A*0101 | A*2601 | B*0801 | B*3801 | CW*0701 | CW*1203 | Pol | DGAANRETKLGKAGYV | ETKLGKAGY |  |  |
| 55 | A*0201 | A*2403 | B*1801 | B*4402 | CW*0501 | CW*1203 | Pol | AETGQETAYFLLKLAG | GQETAYFLL |  |  |
| 18 | A*0201 | A*2402 | B*1402 | B*3901 | CW*0802 | CW*1203 | Pol | GQVRDQAEHLKTAVQM | EHLKTAVQM |  |  |
| 38 | A*2402 | A*6801 | B*1503 | B*4006 | CW*0202 | CW*1502 | Pol | VVPRRKAKIIRDYGKQ | RKAKIIRDY |  |  |
| 20 | A*2403 | A*6801 | B*3503 | B*3801 | CW*0401 | CW*1203 | Pol | GYELHPDKWTVQPIVL |  |  |  |
| 11 | A*0201 | A*0301 | B*0702 | B*4002 | CW*0202 | CW*0702 | Pol | PEKDSWTVNDIQKLVG | not deconv. |  |  |
| 26 | A*0101 | A*2402 | B*3503 | B*5701 | CW*0401 | CW*0602 | Pol | LCKLLRGTKALTEVIP |  |  |  |
| 41 | A*0101 | A*0201 | B*1302 | B*5701 | CW*0602 |  | Pol | LTEEAELELAENREIL | not deconv. |  |  |
| 18 | A*0201 | A*2402 | B*1402 | B*3901 | CW*0802 | CW*1203 | Pol | GQWTYQIYQEPFKNLK |  |  |  |
| 7 | A*0301 | A*0301 | B*3501 | B*5101 | CW*0401 | CW*1402 | Pol | QEPFKNLKTGKYARMR |  |  |  |
| 20 | A*2403 | A*6801 | B*3503 | B*3801 | CW*0401 | CW*1203 | Pol | TGKYARMRGAHTNDVK |  |  |  |
| 7 | A*0301 | A*0301 | B*3501 | B*5101 | CW*0401 | CW*1402 | Pol | ETWWTEYWQATWIPEW |  |  |  |
| 7 | A*0301 | A*0301 | B*3501 | B*5101 | CW*0401 | CW*1402 | Pol | QKTELQAIYLALQDSG |  |  |  |
| 26 | A*0101 | A*2402 | B*3503 | B*5701 | CW*0401 | CW*0602 | Pol | WVPAHKGIGGNEQVDK |  |  |  |
| 34 | A*1101 | A*6801 | B*5301 | B*1518 | CW*0401 | CW*0704 | Pol | LVSAGIRKVLFLDGID |  |  |  |
| 20 | A*2403 | A*6801 | B*3503 | B*3801 | CW*0401 | CW*1203 | Pol | GIRKVLFLDGIDKAQD |  |  |  |
| 53 | A*2902 | A*2902 | B*0702 | B*3503 | CW*0401 | CW*0702 | Pol | WRAMASDFNLPPVVAK | not deconv. |  |  |
| 25 | A*0201 | A*0206 | B*5101 |  | CW*0401 | CW*1402 | Pol | ASDFNLPPVVAKEIVA |  |  |  |
| 18 | A*0201 | A*2402 | B*1402 | B*3901 | CW*0802 | CW*1203 | Pol | HLEGKVILVAVHVASG |  |  |  |
| 26 | A*0101 | A*2402 | B*3503 | B*5701 | CW*0401 | CW*0602 | Pol | VASGYIEAEVIPAETG |  |  |  |
| 26 | A*0101 | A*2402 | B*3503 | B*5701 | CW*0401 | CW*0602 | Pol | KLAGRWPVKTIHTDNG |  |  |  |
| 58 | A*0201 | A*2402 | B*5101 | B*4403 | CW*0401 | CW*1402 | Pol | VESMNKELKKIIGQVR | not deconv. |  |  |
| 19 | A*2402 | A*3002 | B*0801 | B*1801 | CW*0501 | CW*0701 | Pol | AVQMAVFIHNFKRKGG |  |  |  |
| 18 | A*0201 | A*2402 | B*1402 | B*3901 | CW*0802 | CW*1203 | Pol | YRDSRNPLWKGPAKLL |  |  |  |
| 17 | A*1101 | A*3001 | B*3501 | B*1302 | CW*0401 | CW*0602 | Pol | WKGPAKLLWKGEGAVV | not deconv. |  |  |

^1^ "not deconv." denotes minipools where deconvolution was not performed due to limited sample or relatively low T-cell response.
