# Supplementary material for: Mapping HIV-1 Vaccine Induced T-Cell Responses: Bias towards Less-Conserved Regions and Potential Impact on Vaccine Efficacy in the Step Study
Source: PLoS One. 2011 Jun 10;6(6):e20479. doi: 10.1371/journal.pone.0020479 (PMC3112144; doi:10.1371/journal.pone.0020479)
Supplement: Table S2 — Median protein similarity and reactive epitope frequency in subtype B and C strains (DOC) [file pone.0020479.s003.doc]

**Table S2. Median protein similarity and reactive epitope frequency in subtype B and C strains**

|  | Subtype B | |  | Subtype C | |
| --- | --- | --- | --- | --- | --- |
| Protein | Protein homology^a^ | Epitope frequency |  | Protein homology | Epitope frequency |
| Gag | 0.937 | 0.730 |  | 0.829 | 0.062 |
| Nef | 0.775 | 0.233 |  | 0.733 | 0.011 |
| Pol | 0.951 | 0.707 |  | 0.902 | 0.207 |

^a^Protein homology was computed as the percentage of identical amino acid positions between vaccine insert and viral sequences from LANL.
